# The Treatment of Eczema

**Published:** 1908-10-31

**Authors:** 


### DERMATOLOGY.

### THE TREATMENT OF ECZEMA.

In the treatment of eczema the most important fact to be borne in mind is that the lesions must be protected from local irritation. If this principle be strictly adhered to, the affection may be cured by the most simple means. We know that in persons who suffer from eczema there is some predisposing factor which renders the skin highly susceptible to local irritations, but unfortunately we have no exact knowledge of the nature of this predisposing cause. We do know that change of diet, change of climate, or other altered conditions of life may sometimes lead to the spontaneous disappearance of eczema, but generally if we deliberately try to induce this form of cure we fail to produce any effect. It is certain, however, that local irritation is a very important factor in the actual production of eczematous lesions, and we find, in practice, that if the parts can be really protected from local irritants not only will the lesions clear up, but the susceptibility of the skin generally to local irritants will disappear—that is to say, the patient will lose his tendency to eczematous eruptions.

The most fruitful causes of the continuance of eczematous eruptions are (1) the intermittent use of water in the process of the ordinary daily ablutions, and (2) irritation from friction of clothes, and from rubbing and scratching by the patient. By the intermittent use of water the parts are at one time sodden and at another dry, and it is this alternate wetting and drying which is so harmful. On the other hand, one of the best forms of treatment for an acute eczema is the application of a *continuous* wet dressing. In many cases, if the patient can be induced to stay in bed (so that friction by contact with clothes and other objects is avoided) and moist dressing be applied for a few days, the activity of the lesions will at once subside and a rapid cure may be brought about. One of the best means of applying such dressings is to use pads of four or five folds of butter-muslin soaked in a normal saline solution (common salt ʒj. to one pint of boiled water), and well covered with gutta-percha tissue or oiled silk, the dressings to be changed twice a day. Other useful protective applications are the various ointments, pastes, and creams containing oxide of zinc. These may be employed after the wet dressings have been used for a few days, or they may be applied from the first, especially in cases where one cannot keep the patient in bed. The ordinary zinc ointment of the British Pharmacopœia is an efficient application; its value is enhanced by the addition of starch powder to make a paste.* These medicaments must be spread upon strips of butter-muslin and bound on to the parts affected. They should be changed as often as there is any tendency for the dressing to get dry, generally once or twice a day. If the dressing does get dry it must be softened from the outside by dabbing on some olive oil, otherwise it will stick to the lesion and pull away the new-formed epidermis on its removal. A useful addition to the ointment or paste is lenigallol (gr. x.-ʒj. to each ounce of ointment or paste). This substance dries up all the wet points and leaves them covered with little black crusts, which eventually fall off. For very extensive areas it is sometimes more convenient to apply a cream than an ointment. A suitable cream may be made as follows:—

| | | |
|---|---|---|
| Oxide of Zinc | ... ... ... ... | ℥j. |
| Lanolin | ... ... ... ... ... | ʒij. |
| Olive oil / Lime water | } equal parts up to ... | ℥iv. |

Strips of butter-muslin are soaked in this cream and applied.

When an eczema is in a sub-acute stage, or has been got under control by one of the above methods, it is often convenient to apply a protective dressing which will remain on for several days and which will enable the patient to get about without fear of irritation of the lesions by friction, etc. Such a protective dressing is the well-known Unna's paste. The formula for this is:—

| | | | |
|---|---|---|---|
| Gelatin ... ... | 3 | parts | } soak for 10 hours, then heat to dissolve and add |
| Water ... ... | 9 | ,, | |
| Oxide of zinc ... | 2 | ,, | } previously mixed to a paste. |
| Glycerine ... | $4\frac{3}{4}$ | ,, | |

The jelly thus formed is melted for use in a glue-pot or other water-bath, and, when sufficiently melted, it is painted over the affected part. Before it has time to dry, the surface of the coat of paint is mopped over with cotton-wool, which adheres to the paint and forms a kind of felting. Such a dressing may remain on for several days, when it must be peeled off and a fresh one applied. If there is much discharge beneath the dressing this form of treatment is not suitable, and a cream or ointment had better be used until a drier stage is again reached. Other useful applications when there is not much weeping or crusting are the salve-mulls of Unna, particularly the zinc oxide, and the ichthyol and zinc oxide salve-mulls, which act mainly by keeping the lesion well protected from local irritation.

* *i.e.*, Lassar's Paste—Zinci oxidi ʒij., pulv. amyli ʒij., vaselin ℥ss.

